# Assessing Severity of Psychological Distress Among Refugees With the Refugee Health Screener, 13-Item Version

**DOI:** 10.1097/NMD.0000000000000886

**Published:** 2018-09-26

**Authors:** Anna Bjärtå, Anna Leiler, Johanna Ekdahl, Elisabet Wasteson

**Affiliations:** Department of Psychology, Mid Sweden University, Östersund, Sweden.

**Keywords:** Refugee mental health, screening instrument, symptom severity levels

## Abstract

The recent inflow of refugees to Sweden has put pressure on health care as well as revealing a need for methods regarding assessment of refugees' mental health status. The present study investigated the use of the Refugee Health Screener (RHS; Hollifield et al., 2013) to distinguish among severity levels of symptoms of psychological distress in refugees. Refugees residing in asylum accommodations (*n* = 510) were screened with RHS-13, together with screeners for depression, anxiety, and posttraumatic stress disorder (PTSD). Risk for mild, moderate, or severe levels of depression, anxiety, or/and PTSD was used as screening proxy. Receiver operating characteristic analysis rendered cutoff scores of 11, 18, and 25, for mild, moderate, and severe symptoms, respectively. Evaluated against each symptom scale separately, cutoffs performed well. Cutoff 11, previously identified by Hollifield et al. (2016), was also confirmed. However, utilization of additional cutoffs could improve refugee mental health by guiding clinical decision making.

When 2015 came to an end, 65.3 million people worldwide were forcibly displaced from their homes due to conflict, violence, and human rights violations (United Nations High Commissioner for Refugees, 2015). Although the majority is internally displaced, the number of new applications for asylum or refugee status reached record levels in Europe (Eurostat, 2018). Sweden was one of the countries that received most asylum seekers and granted most residence permits (RPs), both regarding population ratio and in total (United Nations High Commissioner for Refugees, 2015). Most refugees have experienced extremely stressful events before, during, and after migration, and a large percentage of refugees worldwide are suffering from stress-related mental health problems (Fazel et al., 2005; Kirmayer et al., 2011). Not only causing individuals great suffering, unrecognized mental health problems may also aggravate integration. There is, for example, a strong association between mental health and employment (*e.g.*, Paul and Moser, 2009), which also is a commonly used indicator of integration. Porter and Haslam (2005) have shown that employment is a key factor for refugee mental health. There is furthermore evidence that interventions provided early on can improve the mental health status of refugees (Murray et al., 2010; Stenmark et al., 2013), thus improving the chances for a successful integration. Put together, early detection of mental health problems among refugees implies great benefits both at the individual and the societal level.

All refugees seeking asylum in Sweden are offered a routine health screening (Socialstyrelsen, 2015). The health screening includes a checklist of items concerning mental health, such as trouble sleeping, psychological problems, drug abuse or experiences of war, torture, separation, or imprisonment. However, a structured assessment of mental health has not been included in the health screening, and there is a call for efficient instruments that can help health care personnel identify symptoms of mental health issues. Michael Hollifield and his colleagues (Hollifield et al., 2013, 2016) have developed a screening tool, the Refugee Health Screener (RHS), to assess symptoms of disorders common among refugees (*i.e.*, anxiety, depression, and posttraumatic stress disorder [PTSD]). The RHS was designed to be a brief and culturally responsive first screener. It has been translated into 17 different languages and validated within several different refugee populations and is therefore comprehensible and applicable to many different groups of refugees. Due to its brevity and specific aim, the RHS could be ideal for inclusion in the routine health screening.

The RHS is not constructed as a diagnostic tool but as a highly sensitive first screening tool to identify individuals suffering from mental health problems (PTSD: sensitivity 0.81/specificity 0.87, anxiety: 0.94/0.86, and depression: 0.95/0.89; Hollifield et al., 2013). A cutoff 12, for the 15-item version, or 11, for the 13-item version (Hollifield et al., 2016), has been suggested to identify people in need for further assessment. However, Hollifield et al. (2016) acknowledges that clinical sites might need to adapt the cutoff in accordance with the service and resources available. Due to the recent high influx of refugees, the capacity to handle health issues is limited. Cutoffs that could distinguish between symptom severity levels would be very helpful in guiding health care personnel prioritize among patients.

Many screening instruments for measuring psychological distress of various kinds have so-called clinical cutoffs to identify severity of symptoms. For example, the Patient Health Questionnaire nine-item scale (PHQ-9; Kroenke et al., 2001) and the Generalized Anxiety Disorder seven-item scale (GAD-7; Spitzer et al., 2006) are two screening instruments commonly used to indicate if symptoms of depression or anxiety, respectively, ought to be considered mild, moderate, or severe. A screening resulting in moderate or severe symptoms is considered relevant in a clinical context, meaning that the risk of having a diagnosis that ought to be treated by a specialist is significant, whereas people reporting mild symptoms may serve from a smaller preventive treatment. Being able to differentiate between individuals that might benefit from lighter interventions and those in need of extensive care improves the utility of an instrument and can help guide clinical decision making.

The purpose of the present study was to investigate if the RHS-13 could be used as a screener to distinguish among severity levels of symptoms of psychological distress. The desired cutoffs are one for mild symptoms indicating subclinical levels of distress, one for moderate indicating clinically significant levels, and one for severe as a strong indicator of a psychiatric diagnosis. If the RHS-13 can be used as a graded screening instrument, the mental health status of refugees could be assessed and managed in a more efficient and cost-effective way. Thus, to enable a more fine-tuned assessment of mental health problems among refugees, the present study aims to identify and evaluate the application of cutoffs assessing symptom severity for the RHS-13. Operating characteristics for mild, moderate, and severe symptoms of the RHS were derived using PHQ-9, GAD-7, and the primary care PTSD (PC-PTSD; Prins et al., 2003) as screening proxy criteria. We thereafter investigated the cutoffs regarding their predictive value and their ability to differentiate between symptoms of depression, anxiety, and PTSD. Furthermore, the cutoffs were investigated regarding their sensitivity to contextual factors, such as status of asylum application.

## METHODS

The present study is part of the AMIR project (assessment of mental health and early intervention for refugees), aiming to develop a model for early assessment and treatment of mental health problems among refugees. The regional ethical review board has approved the full project.

### Sample Frame and Procedures

The sample was a cohort of refugees 18 years or older living in facilities provided by the Swedish migration agency. The sample consisted of both individuals seeking asylum in Sweden and those who recently had been granted asylum, awaiting a transfer decision from the facilities to a municipality. The screening took place in the region Jämtland/Härjedalen, Sweden, during the period of November 2016 to April 2017. All materials were translated into the five most common language groups of the region (Arabic, Dari, Farsi, Somali, and Tigrinya) and also into English and Swedish. Participants were recruited by an information letter sent by regular mail to all refugees in the region speaking any of above languages (*n* = 1265). The letter included a schedule of the screening tour. Advertisements were posted at asylum facilities in advance of each screening. Participants could take the survey during a screening occasion at or nearby their housing facility or online through a Web address. The survey was produced in Qualtrics software (Qualtrics; Provo, UT, 2005), which is a high security online survey system.

Together with bilingual experts, we visited 12 different asylum housings and 9 other meeting points for refugees (such as the Red Cross or language training meetings) at 26 different occasions. People interested in participating were asked to take the survey on-site using the Qualtrics application on tablets (iPad Air 2). Audio support was available in Arabic, Dari, Farsi, and Tigrinya for individuals with low reading proficiency. Bilingual staff members supported with instructions and clarification to people in need. A second information letter was sent to all asylum seekers in the region, 18 years or older (*n* = 1332), after the screening tour. In that letter, we offered people speaking other languages and people who had missed the on-site screening opportunities to take the survey online. We clarified that people who had already completed the survey should not take it again.

A total of 577 responses were collected, of which 18 individuals had used audio support (11 Dari, 4 Arabic, 2 Farsi, and 1 Tigrinya), and only 15 completed the survey online. Sixty-seven respondents did not complete the survey and were therefore excluded from the dataset. Of the remaining 510 respondents, 250 completed the survey in Arabic, 192 in Dari, 35 in Farsi, 18 in Tigrinya, 5 in Somali, and 10 in English, including all respondents with sound support and all online responses (see Table 1 for further information about the remaining sample).

**TABLE 1:**
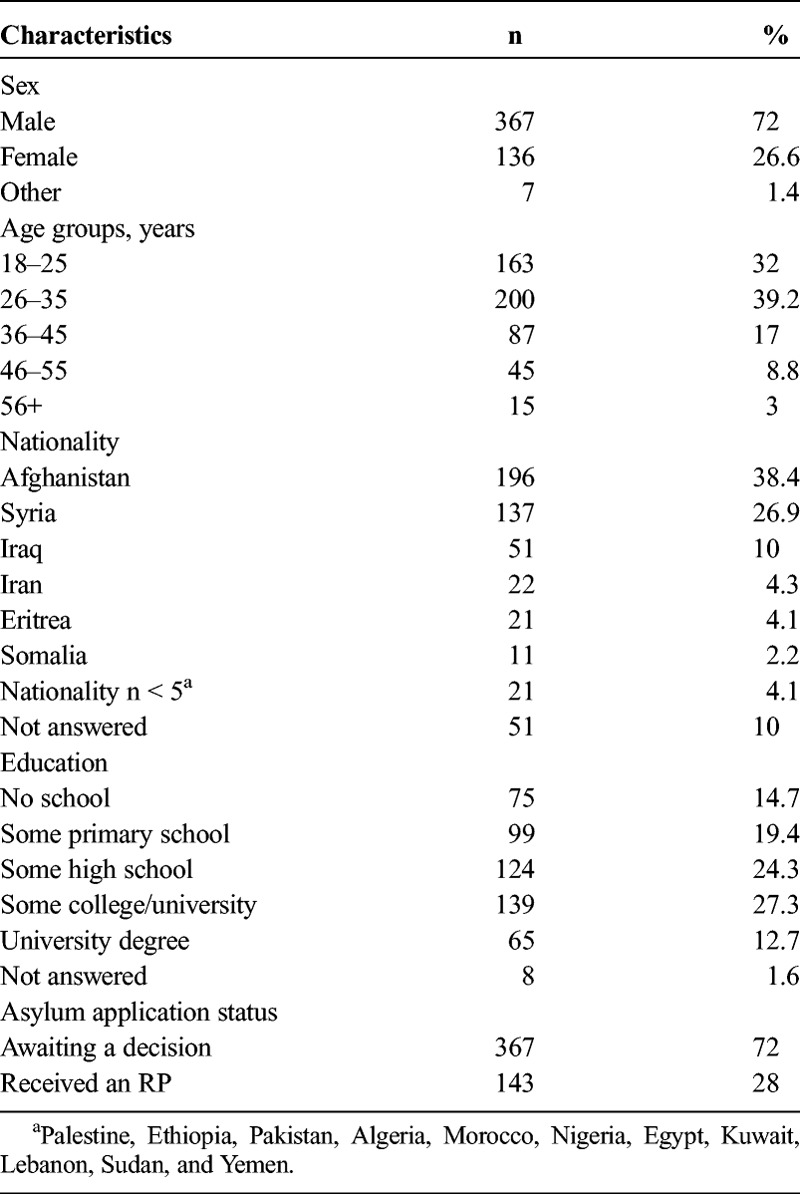
Demographic Characteristics From 510 Respondents

### Instruments

Beyond sufficient psychometric properties, instruments were selected based on criteria of importance for the specific population. They should, for example, be brief, simple to understand, and culturally responsive. Two instruments with cutoffs measuring symptom severity were used as screening proxy criteria for mild, moderate, and severe levels of depression (PHQ-9) and anxiety (GAD-7). PTSD, however, is a complex disorder and there is a large variation in expression of symptoms. To our knowledge, there are no valid screening instruments with specified cutoffs used to determine symptom severity. There are, however, several instruments with dichotomous cutoffs indicating risk of having a PTSD diagnosis. We used a brief instrument, the PC-PTSD-4, which has been used to identify both subclinical and clinical symptoms. A measure of quality of life, the World Health Organization's Quality of Life–brief instrument (WHOQOL-BREF; WHOQOL Group, 1998), was also used as a validity check primarily for the Dari version of RHS, which was translated for the present study.

Our bilingual staff translated all materials used in the study (information letters, advertisement, informed consent, etc.). Any instrument that needed translation was translated by a rigorous process, similar to the World Health Organization's guidelines (Guidelines for Translation; World Health Organization, n.d.) and in consensus with the developers of the RHS, to obtain semantic and cultural equivalence (*e.g.*, Ekrut, 2010). Professional translators conducted the forward translation based on written instructions from the authors. Translations were then reviewed and adjusted, if needed, by bilingual personnel employed in the project. The bilingual staff consisted of educated individuals with health care education, native in the target language and with a high proficiency in either English, Swedish, or both languages. All translated materials, including information letters and instruments, were then discussed in focus groups consisting of monolingual individuals from the target group and the bilingual project personnel. Instruments were then back-translated by another professional translator (in some cases also by another independent bilingual person), and adjustments (if needed) were conducted by the bilingual personnel together with the research group. Translations of the RHS existed in all of the target languages except for Dari. For PHQ and GAD, translations in English, Arabic, and Swedish existed, and for PC-PTSD, only English and Swedish.

#### The Refugee Health Screener, 13-Item Version

The Refugee Health Screener, 13-Item Version (RHS-13) has been developed to screen for psychological distress among refugees (Hollifield et al., 2013). The development of the scale is based on symptoms relating to PTSD, anxiety, and depression. The original version has 15 items with excellent internal consistency (Cronbach's α = 0.92). However, a 13-item version of the scale has shown similar psychometric properties. (Hollifield et al., 2016). By removing two items with low factor loadings, the internal consistency was strengthened (*α* = 0.96) without compromising the concurrent and predictive validity of the scale (sensitivity range, 0.82–0.96 and specificity range, 0.86–0.91 at cutoff ≥11 for diagnostic proxies for PTSD, depression, and anxiety). The scale's 13 items are answered with a five-point Likert scale (0–4; total range of scores, 0–52). Each point is not only labeled with text (not at all, a little bit, moderately, quite a bit, extremely), they are also numbered (0–4) and visually represented with a bottle filled to different degrees (from empty to full). The sum of scores is used, and recommendation for screening purposes is a cutoff of 11.

#### The Patient Health Questionnaire 9

The Patient Health Questionnaire 9 (PHQ-9) is a dual-purpose instrument that can be used to establish depressive disorder diagnosis and also to grade symptom severity (Kroenke et al., 2001). The scale has nine items with a four-point Likert scale (0–3; range, 0–27) and labeled alternatives (not at all, several days, more than half the days, nearly every day). The instrument has shown a good internal consistency (Cronbach's α = 0.89; test-retest reliability, 0.84). Both construct and diagnostic criterion validity have been established in several studies with sufficient sensitivity (0.71–0.87) and specificity (0.88–0.95; Gilbody et al., 2007). The sum of scores is used, and scores of 5, 10, 15, and 20 have been recommended as cutoffs screening for mild, moderate, moderately severe, and severe symptoms, respectively. A cutoff of 5 has shown efficient in identifying subthreshold depression, 10 a spectrum of depressive disorders including major depression, while scores above 15 have shown to be highly efficient in identifying major depression. For the purposes of the present project, we used cutoffs 5, 10, and 15 to identify mild, moderate, and severe symptoms on the RHS-13.

#### The Generalized Anxiety Disorder 7

The Generalized Anxiety Disorder 7 (GAD-7) is an instrument originally developed to screen for generalized anxiety disorder (Spitzer et al., 2006). It has, however, frequently been used to assess severity of anxiety symptoms more generally in primary care settings (Kroenke et al., 2007). The scale has seven items, scored in the same way as PHQ-9 (see previous description; range, 0–21). It has shown highly reliable (Cronbach's α = 0.92; intraclass correlation, 0.83) and seems to function well as an indicator of symptom severity. The sum of scores is used, and cutoffs of 5, 10, and 15 have been recommended for mild, moderate, and severe symptoms, respectively. However, a cutoff of 8 has shown to increase sensitivity in identifying clinical levels of several anxiety disorders (Kroenke et al., 2007). For the purposes of the present project, we used cutoffs 5, 8, and 15 for mild, moderate, and severe symptoms, respectively.

#### The Primary Care PTSD-4

The Primary Care PTSD-4 (PC-PTSD-4) was developed as a screener for PTSD in primary care settings (Prins et al., 2003). The scale has four items (a five-item screener has been released after the start of the project; Prins et al., 2016) asking about four characteristic symptoms (intrusion, avoidance, hyperarousal, emotional numbing) related to any traumatic event. Although being very brief, it has shown to be highly efficient in identifying PTSD diagnosis, and it performs equally well to other more lengthy and thorough scales, such as the PCL (Bliese et al., 2008). Items have “yes” or “no” responses, and the number of “yes” are summed up. Scores 2 or higher are considered to be at subthreshold levels (sensitivity range from 0.85–0.91 and specificity range from 0.71–0.72; Bliese, 2008; Prins et al., 2003), and scores 3 or higher at a clinical level (sensitivity, 0.76–0.78 and specificity, 0.87–0.88). Scores of 4 have shown a very high specificity (0.93–0.96) but a rather low sensitivity (0.41–0.54). Thus, for the purpose of the project, we have used 2, 3, and 4 for mild, moderate, and severe symptoms, respectively.

#### The World Health Organization Quality of Life–Brief Version

The World Health Organization Quality of Life–Brief Version (WHOQOL-BREF) is an instrument developed by the World Health Organization as a transcultural measure of quality of life and health in four domains: physical, psychological, social relationships, and environmental health (WHOQOL Group, 1998). The instrument is a brief version of the WHOQOL-100 (WHOQOL Group, 1995). It has 26 items with labeled alternatives, scored 1 to 5. Raw scores are transformed to domain scores ranging from 4 to 20. In a large cross-sectional study, carried out on the general population in 23 different countries, sufficient reliability and validity were established. Cronbach's α was acceptable for the total sample (0.82 for physical health, 0.81 for psychological health, 0.68 for social health, and 0.80 for environmental health), and the scale discriminated well between people that were ill and well. Moreover, both global and specific (for all countries) analyses were performed, showing a good cross-cultural application of the scale.

### Data Treatment and Analysis

As mentioned previously, data from participants that did not complete the survey were removed from the analysis. We treated instruments in accordance with recommendations from scale developers (see previously). Dummy variables were created for all cutoffs on PHQ-9, GAD-7, and PC-PTSD-4, respectively. Thereafter, proxies were compiled into a “mental distress proxy index” indicating any (of the three) symptoms, accordingly to if a person showed symptoms on one or more of the proxies. For example, to be coded as having mild levels of distress on the proxy index, a person ought to have scored at least 5 on PHQ-9, and/or GAD-7, and/or at least 2 on PC-PTSD-4. The same was made for moderate (cutoff scores 10, 8, and 3 on above scales, respectively) and severe levels (15, 15, and 4). This procedure was used because RHS is designed to identify symptoms of mental distress, and not of a specific diagnosis. Therefore, we base the analysis on the ability of RHS to assess any symptom of the three categories of symptoms that the development of RHS is based on.

A descriptive analysis of the scales is followed by a brief evaluation of the Dari translation, which is the new RHS translation conducted for the present project. The largest language groups of the sample were Arabic and Dari. This enabled a comparison between the two languages regarding internal consistency (with Cronbach's α) and convergent validity (correlation between RHS and the symptoms, and quality of life scales). Sample sizes from the other language groups did not allow for proper analyses; however, data for the whole sample is presented.

Receiver operating characteristics (ROC) analysis was conducted to determine cutoffs for RHS, using each of the indexed symptom levels (mild, moderate, and severe, respectively). The area under the ROC curve (AUC) indicates a test's performance by indexing the full range of operating characteristics for diagnostic accuracy in a tool (*e.g.*, Zweig and Campbell, 1993). In the work with obtaining cutoffs, we aimed for as high a sensitivity and specificity as possible for the mild level. Therefore, we used maximization of sensitivity and specificity for the first cut point. For the moderate and severe levels, we were aiming for as high specificity as possible not reducing sensitivity below 70%.

Psychometric properties of the new cutoffs were thereafter calculated, and false-negatives for the mild level were inspected. Prevalence based on the new cutoffs were calculated for the whole sample, and also split by asylum status. Because many of the refugees we met had already received a positive decision and were waiting for transfer from accommodations, we investigated whether levels of distress differed between people with different asylum status using Pearson's χ*^2^* and adjusted standardized cell residuals. Mean ratings were compared with an independent *t*-test. Finally, the new cutoffs' ability to discriminate levels of symptoms on the respective symptom scales was analyzed with a one-way analysis of variance (ANOVA) for each scale. All pairwise comparisons were Bonferroni corrected.

## RESULTS

### Descriptive of Scales

Overall high mean scores were found on all scales with the mean of RHS-13 at 22.9 (SD, 13.5), 11.5 (6.9) for PHQ-9, 9.4 (6.2) for GAD-7, and 2.5 (1.4) for PC-PTSD-4. All symptom scales ranged from the minimum to the maximum score of each scale. The quality of life measures showed mean values of 13.4 (3.4), 12.3 (2.9), 12.8 (4.0), and 10.8 (3.0), for the physical, psychological, social relations, and environmental domains, respectively. A mean score of 12 for the respective domain has been identified as a midpoint where quality of life is assessed as neither good nor bad (Skevington et al., 2004).

### Reliability and Validity

The internal consistency was excellent for both the Arabic and Dari version of the RHS-13, with Chronbach's *α* of 0.92 and 0.91, respectively, and *α* = 0.92 for the total sample. The RHS-13 showed significant moderate to very strong positive correlations with all symptom measures (*r* = 0.44 to 0.82) and significant weak to moderate negative correlations with all domains of the WHOQOL-BREF (*r* = −0.24 to −62) for both languages, with slightly weaker coefficients within the Dari group (all *p* < 0.001, see Table 2). The pattern is coherent over both groups, and for the total sample.

**TABLE 2:**
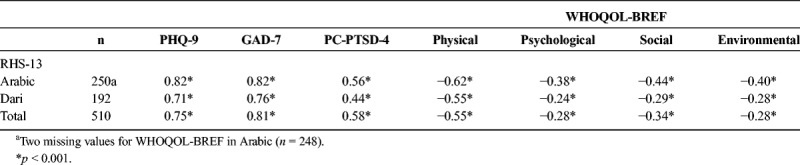
Pearson *r* for Correlations Between RHS-13 and the Symptom Measures as well as Quality of Life Domains for the Arabic- and Dari-Speaking Groups, and for the Total Sample

### ROC Analysis

The AUC for the mild level (test variable: RHS-13, state variable: proxy index for mild levels of distress) showed high concordance between the tests, with AUC of 0.949 (*p* < 0.001; *SE* = 0.013; 95% confidence interval [CI], 0.923–0.975). Maximization of sensitivity and specificity rendered a cut score of 11. This is the same cutoff score previously identified by Hollifield et al. (2016), also validated by Kaltenbach et al. (2017). Sensitivity and specificity are in ranges found previously (see Table 3 for psychometric characteristics of the respective cutoffs). ROC analysis for the moderate cutoff showed an AUC of 0.915 (*p* < 0.001; *SE* = 0.015; 95% CI, 0.885–0.945), with the highest specificity for at least 70% sensitivity at cutoff 18. Finally, the analysis for the severe cutoff showed an AUC of 0.855 (*p* < 0.001; *SE* = 0.016; 95% CI, 0.823–0.887), with the highest specificity for at least 70% sensitivity at cutoff 25.

**TABLE 3:**
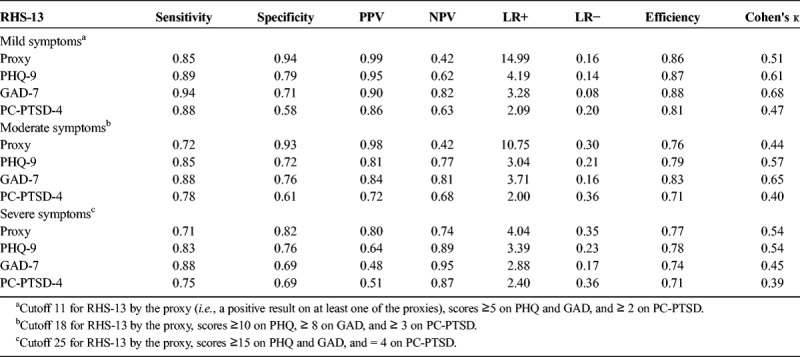
Screening Utility of the Identified Levels of Mild, Moderate, and Severe Symptoms of Mental Distress for RHS-13, by the Screening Proxy Index and Each Screener Separately

### Psychometric Properties of the Cutoffs

As shown by Table 3, the lower cutoff shows satisfactory metrics for identifying any (of the three) mild symptoms. The weak negative predictive value (NPV) was due to a high rate of false-negatives (69) in relation to the true negatives (50) in the present sample. Mean values of the false-negatives were 5.83 (SD, 3.49) for PHQ-9, 3.71 (2.47) for GAD-7, and 1.90 (1.37) for PC-PTSD-4. The mild level of RHS-13 missed to detect a total of 35 individuals with clinically significant symptoms (*i.e.*, moderate symptoms and more), of which 10 individuals rated at severe levels on one of the scales. Likelihood ratios show a good screening accuracy for any symptom, at both the mild and the moderate level. Each symptom scale, separately, shows satisfying sensitivity at all levels. Effect sizes are sufficient and show a good quality.

### Prevalence and Ability of Cutoffs to Discriminate Levels of Mental Health by Symptoms

Seventy-seven percent of the respondents had scores of 11 and above (61% scored 18 and above, and 45% scored 25 and above). This is a higher prevalence compared with previous findings (Hollifield et al., 2016; Kaltenbach et al., 2017). However, a division between individuals that had received a positive decision (RP) and those still awaiting decision (ND, no decision) gave an explanatory and reasonable picture. The prevalence of severe symptoms were more than twice as high among NDs (52%) than RPs (25%, adjusted standardized residuals *z* = ±5.48; χ*^2^*(3) = 39.83; *p* < 0.001). In the other end, the prevalence of no and mild symptoms were twice as high among RPs (36% and 24% for no and mild symptoms, respectively) than NDs (18% and 13%, *z* = ±4.34 and ±3.14 for no and mild symptoms, respectively; see Table 4). Mean scores differed significantly (mean, 16.7; SD, 11.7, and mean, 25.3; SD, 13.5, for RP and ND, respectively; *t*(508) = 6.69, *p* < 0.001, *d* = 0.68).

**TABLE 4:**
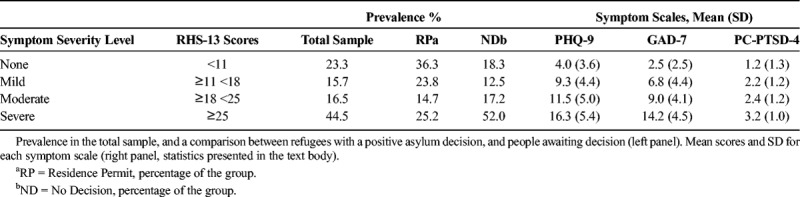
Prevalence of Mental Distress Based on the New Cutoffs for RHS-13

As shown by significant ANOVAs for both PHQ-9 (*F*(3,506) = 176.37, *p* < 0.001, *η^2^* = 0.51) and GAD-7 (*F*(3,506) = 237.76, *p* < 0.001, *η^2^* = 0.59), mean ratings increased significantly for each severity level of RHS (*p* = 0.024 and 0.004 between mild and moderate symptoms for PHQ-9 and GAD-7, respectively, all other *p* < 0.001). A significant ANOVA was also found for PC-PTSD-4 (*F*(3,506) = 105.72, *p* < 0.001, *η^2^* = 0.32). However, the mild and moderate levels did not differ (*p* < 0.001 for all other comparisons).

## DISCUSSION

The results show that the RHS-13 is highly efficient in identifying symptoms of mental distress related to anxiety, depression, and posttraumatic stress. The present study validates the previously identified cutoff of 11 (Hollifield et al., 2016) as a highly sensitive cutoff, here for mild symptoms. We also suggest the use of a second cutoff, at 18 or above, to identify clinically significant (*i.e.*, moderate) symptoms that ought to be further assessed. Furthermore, a third cutoff, at 25 or above, could be used to identify individuals in a more acute need of advanced care. Further results from the AMIR project show that most individuals with severe symptoms, attending further assessment, had mental health problems at diagnostic levels. This utilization could save both time and money for all involved parts and, foremost, decrease suffering and increase the quality of life for refugees.

The cutoffs derived from the proxy index differentiate psychological symptoms and correspond well to the cutoffs used for GAD-7 and PHQ-9. However, differences between the mild and moderate symptom levels are smaller to none (for PTSD), indicating that further work can be done to test cutoffs. As shown throughout the analysis, results from the PC-PTSD-4 are a bit sparser than the other two measures. This is likely due to the very brief instrument rendering insufficient variation in scores related to level of distress, hence the result. Given also that a higher specificity in the severe level would have been desirable, it is possible that higher scores for both the moderate and severe cutoffs could be more feasible. They are, however, based on criteria of other screening instruments that have shown to be highly efficient in identifying the levels we aimed for (*i.e.*, PHQ-9 and GAD-7).

The severity levels of RHS also seem to be sensitive to change. The difference between people awaiting asylum decision and those with a positive decision shows stunning results with prevalence of severe symptoms twice as high among people awaiting decision (52% *vs*. 25% for ND and RP, respectively) and the opposite relation in the no and mild symptom range. Although incidence was lower among refugees that had received a positive decision, 64% met the criteria for mild symptoms (*i.e.*, scores 11 and above). This estimate is higher than previously found. For example, using a cutoff of 11 for RHS-13, Hollifield et al. (2016) found a mean prevalence of 38% with the largest incidence among Iraqi refugees (58%), and Kaltenbach et al. (2017) found a prevalence of 42% for the self-administered version of RHS-13. The higher estimates found in the present study could be explained by the fact that refugees remained in housing facilities under the same conditions as asylees. That is, even if people had received a positive decision, they were still living in facilities awaiting decision for transfer. Living conditions in asylum housings are also reflected by relatively low mean scores of the environmental domain of the WHOQOL-BREF (mean, 10.8; SD, 3.0). It could also be related to premigration regional and demographic factors. For example, many refugees from Syria, which is a heavily war-affected region, are granted RP (Migrationsverket, 2018). To be clear, it was explicitly stated that no one other than researchers in the project had access to data and that how participants responded could not in any way affect the decision of their asylum application. Thus, the high prevalence of severe symptoms in the group still awaiting their asylum decision should not be due to overreporting or malingering for any secondary gains.

Limitations of the study are the population per se. In addition to being a group where the majority has a background of extremely difficult experiences, they are also living under a lot of pressure, with a highly unstable situation and substandard conditions. Consequentially, and naturally, this leads to extremely high prevalence of mental distress and low scores regarding, for example, living conditions, which causes a smaller variation in the dataset. Moreover, a pilot study of the translated versions of all scales could have been performed before the final study. However, the refugee situation in 2015 and 2016 called for urgent measures and focus group discussions of all materials were used instead. Another limitation is the proxies. We are well aware that a screening instrument is not a criterion standard and that diagnostic probability rates likely are lower. A diagnostic interview would certainly have been a much stronger validation. However, given previous psychometric validation of the proxies, we do believe that this procedure has rendered cutoffs that can be of valuable use to clinicians and other people working with refugee health.

## CONCLUSIONS

The results from the present study further develop the work by Hollifield and his colleagues. By providing cutoffs that can be used to determine severity levels of the distress, health care personnel can be guided in clinical decision making for adequate interventions. Hopefully, this will lead to better and more efficient care taking of refugees experiencing mental health problems and, at the same time, a cost-effective use of public resources.
